# *QuickStats:* Age-Adjusted Rates* of Suicide,^†^ by State— National Vital Statistics System, United States,^§^ 2017

**DOI:** 10.15585/mmwr.mm6836a5

**Published:** 2019-09-13

**Figure:**
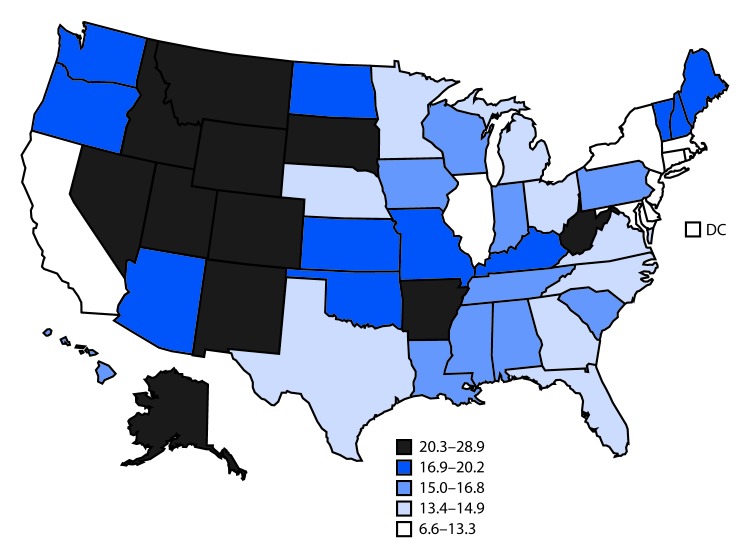
In 2017, the U.S. age-adjusted suicide rate was 14.0 per 100,000 population, but rates varied by state. The five states with the highest rates were Montana (28.9 deaths per 100,000 population), Alaska (27.0), Wyoming (26.9), New Mexico (23.3), and Idaho (23.2). The five with the lowest rates were the District of Columbia (6.6), New York (8.1), New Jersey (8.3), Massachusetts (9.5), and Maryland (9.8).

**Source:** National Center for Health Statistics, National Vital Statistics System. Mortality Data, 2017. https://www.cdc.gov/nchs/deaths.htm.

For more information on this topic, CDC recommends the following link: https://www.cdc.gov/violenceprevention/suicide/index.html.

